# Mental Health of Nurses during the Fourth Wave of the COVID-19 Pandemic in Poland

**DOI:** 10.3390/ijerph19031785

**Published:** 2022-02-04

**Authors:** Beata Dziedzic, Ewa Kobos, Zofia Sienkiewicz, Anna Idzik

**Affiliations:** Department of Development of Nursing, Social and Medical Sciences, Faculty of Health Sciences, Medical University of Warsaw, Żwirki i Wigury 61, 02-091 Warszawa, Poland; ekobos@wum.edu.pl (E.K.); zofia.sienkiewicz@wum.edu.pl (Z.S.); aidzik@wum.edu.pl (A.I.)

**Keywords:** nurses, mental health, anxiety, depression, stress, COVID-19

## Abstract

In the face of the current COVID-19 pandemic crisis, healthcare professionals, including nurses who provide direct care for patients, are at particular risk of mental health problems. The aim of the study was to evaluate the prevalence of symptoms of depression, anxiety, and stress among nurses working in healthcare facilities during the COVID-19 pandemic. Materials and methods: This was a cross-sectional observational study. A total of 333 professionally active nurses participated in the study. Data was collected in the period from 10 November to 20 November 2021. We collected sociodemographic data and used the short form of Depression Anxiety Stress Scale (DASS-21) to assess the mental health among nurses. Results: Severe and very severe symptoms of depression were found in 23.1% of nurses, whereas moderate symptoms were detected in 30.3%. High to very high levels of anxiety were observed in 46.5% of respondents, while 25.8% of nurses showed a moderate level of anxiety. Moderate and high levels of stress were found in 35.4% and 14.1% of the respondents, respectively. Contact with a patient suspected of having SARS-CoV-2 infection was a significant predictor of depressive symptoms. Gender, workplace, and contact with patients suspected of SARS-CoV-2 infection and patients with COVID-19 were significant predictors of anxiety, whereas contact with patients suspected of being infected with SARS-CoV-2 and COVID-19 patients was a significant predictor of stress. Conclusions: High scores for depressive symptoms, anxiety, and stress among Polish nurses during the fourth wave of the COVID-19 pandemic are indicative of a direct threat to the mental health of nurses. Targeted support strategies need to be developed and implemented to prevent the deterioration of mental health in this group.

## 1. Introduction

The outbreak of the COVID-19 pandemic and the spread of SARS-CoV-2 have had an impact on the functioning of societies around the world [1] and have been the source of significant mental stress [2]. This may particularly concern health care workers, including nurses, due to their direct contact with and care of patients, which significantly increases the risk of SARS-CoV-2 infection and developing COVID-19 [3]. Organisational changes at work, work overload, lack of or inadequate amount of appropriate equipment, and personal protective equipment (PPE) may be an additional burden for health care workers [4,5]. Additionally, medical staff are concerned about transmitting the virus to their families, as well as about their own safety [2,6]. There is evidence that the nursing profession is associated with a significant increase in psychological problems, including higher levels of stress, anxiety, and depressive symptoms, compared to other healthcare professions [6,7,8].

Providing an appropriate level of healthcare is not possible without the good physical and mental health of nurses [9]. Nurses are one of the key professionals involved in the fight against the pandemic [10] and have been called upon to meet global public health needs during the COVID-19 crisis [11]. They are directly involved in providing medical care for patients in both hospital and home settings [12]. According to WHO data, nurses account for 59% of healthcare workers globally and 57% in Europe [10]. In Poland, according to the data of the Supreme Council of Nurses, 231,612 nurses were employed in December 2020, which is also the largest professional group of healthcare workers [13]. Thus, nurses are the main force in preventing health system collapse during the COVID-19 pandemic and are the main group involved in the care for infected patients [14].

Furthermore, medical professionals emphasise their physical and mental exhaustion that is not only related to caring for patients, but also to the necessity to make difficult decisions and the distress resulting from the death of patients and colleagues [5,15]. The issue of mental health in healthcare professionals has been moved to the centre of attention of international organisations. The WHO report expressed concerns about the mental health of healthcare workers who are exposed to high-demand settings during the ongoing pandemic. The data from this report show that healthcare professionals working at the frontline experience higher levels of anxiety (13%) and depression (12.2%) compared to non-medical professionals, where anxiety and depression are experienced by 8.5% and 9.5% of people, respectively. Overall, 43% of healthcare professionals, including 27% of nurses, have experienced anxiety. In the proposed strategy, the experts paid particular attention to the activities related to the policy and management of healthcare professionals in terms of planning support for medical personnel, as well as promoting and protecting their mental health. They pointed out that collecting data on the well-being, stress levels, and mental state of employees, followed by providing specialist psychosocial assistance if needed, should be one of the elements of this strategy [16]. Cai et al. also reported that nurses felt more anxious and nervous when fulfilling their duties compared to other professionals (mean score of 1.42 ± 0.87 for nurses vs. 1.18 ± 0.78 for doctors, 1.19 ± 0.84 for medical technicians, and 1.20 ± 1.30 for other hospital personnel) [6]. Important data were reported in a meta-analysis by Al Maqbali, where the prevalence of depression, anxiety, and stress symptoms among nursing staff was 35%, 37%, and 43%, respectively [17]. 

Identification of the most vulnerable medical professionals is important from the point of view of their further functioning [18,19,20] and the negative impact on patient care, leading to its reduced quality [7]. Therefore, this screening study assessed the mental health of nurses who have been struggling to provide patient care for over a year and a half during the pandemic. Drawing attention to the psychological well-being of nurses will allow for developing a support strategy and thus improving the level of care. Furthermore, the results of this study can serve as a benchmark for designing future research.

## 2. Methods

### 2.1. Aim

The aim of the study was to evaluate the incidence of symptoms of depression, anxiety, and stress among nurses working in healthcare facilities during the COVID-19 pandemic.

### 2.2. Participants and Data Collection 

This was a cross-sectional observational study. Data was collected in the period from 10 November to 20 November 2021. At that time, over 3.5 million people were infected in Poland from the beginning of the pandemic (4 March 2020), including approximately 89 thousand fatalities. Daily cases of infection in this period were estimated at about 18.000–23.000 [21]. The Polish version of the questionnaire was created using Google Forms. It was posted on nursing websites and sent by e-mail to all nurses on the contact list who were employed in healthcare facilities. Nurses were also asked to further disseminate the questionnaire by sending it to other working nurses in order to reach the widest possible group of respondents. It is difficult to estimate the number of nurses active in Internet forums. A total of 248 questionnaires were returned. A total of 85 questionnaires were sent to the nurses on the contact list and all were returned. Ultimately, 333 completed questionnaires were included in the analysis. The inclusion criteria for the study were age ≥ 18 years, professional activity in healthcare facilities, and informed consent to participate in the study. The sample size for this study, calculated for 95% confidence interval and 0.5 fraction size with a maximum estimation error of 5%, was 384.

### 2.3. Research Tools

#### 2.3.1. Sociodemographic Data

The characteristics of the study group included the socio-demographic variables, such as gender, age, place of residence, education, professional seniority, place of work, and the frequency of contact with patients with suspected or confirmed SARS-CoV-2 infection.

#### 2.3.2. Screening Questionnaire (DASS-21)

The Depression Anxiety Stress Scale (DASS), developed by Lovibond and Lovibond in 1995 [22], was used to assess the mental health of nurses (symptoms of depression, anxiety, and stress). Two versions of DASS are offered (42- and 21-item), with the 21-item version consisting of a subset of items from the 42-item version [23]. This study was conducted using the short version of DASS-21 in the Polish translation by Professor Makara-Studzińska in cooperation with Petkowicz, Urbańska, and Petkowicz [24]. The psychometric properties of the Polish version of DASS-21 [25] were determined in a sample of 212 medical students, with the obtained results indicating good internal consistency. For the overall score in the scale, the Cronbach’s alpha accuracy was 0.93. For the individual subscales: 0.86 for the depression subscale, 0.80 for the anxiety subscale, and 0.86 for the stress subscale. Internal consistency for this study according to Alpha Cronbach was 0.94 for the overall score, 0.89 for the depression subscale, 0.86 for the anxiety subscale, and 0.83 for the stress subscale.

The scale consists of 21 items divided into three subscales (depression subscale—7 questions, anxiety subscale—7 questions, stress subscale—7 questions). Answers on the prevalence of symptoms during the last week are rated on a 4-point Likert scale from 0 to 3 (0—never true for me, 3—very true for me most of the time). Interpretation for the individual subscales is as follows: severity of depression: 0–9 normal, 10–13 mild, 14–20 moderate, 21–27 severe, >28 very severe; anxiety: 0–7 normal, 8–9 mild, 10–14 moderate, 15–19 severe, >28 very severe; stress: 0–14 normal, 15–18 mild, 19–25 moderate, 26–33 severe, >34 very severe. The scale is a tool used to assess mental health in the last 7 days. It can be used as a screening tool and the result is not a clinical diagnosis [22,23].

### 2.4. Data Analysis

The normality of the distributions was assessed with the Shapiro-Wilk test. The Mann-Whitney U test was used to assess the gender-related differences between the groups. ANOVA was used for more than two groups, for independent samples. Multiple regression analysis was performed for all potential predictors. When applying the criterion for constructing regression models based on the sample size, the maximum number of predictors was set at 7. Explanatory variables are included in the analysis. Adjusted R2 was used in the regression model. According to the previously adopted criteria, all variables had a distribution consistent with the normal distribution. Outliers were eliminated each time in the process of building regression models. The results were considered statistically significant if the value of the calculated test probability was *p* ≤ 0.05. SPSS (Poland) was used for calculations.

## 3. Results

A total of 333 participants took part in the study, including 97.6% of women and 2.4% of men. Most of the respondents (35.5%) were aged 20–30 years and came from urban agglomerations with a population of over 100,000 (60.1%). The length of service <5 years was declared by 35.1% of respondents, while 26.7% of respondents reported professional work experience of 21–30 years. More than half of respondents (50.5%) worked in a hospital (non-infectious ward), while 26.4% and 3.6% of respondents were employed in a hospital transformed into an infectious disease hospital and in an infectious disease hospital, respectively, whereas 12.3% of nurses worked in an outpatient clinic. Most of the respondents (54.6%) reported very frequent and frequent contact with patients suspected of SARS-CoV-2 infection. Sporadic contact with COVID-19 patients was declared by 43.8% of the surveyed nurses, while very frequent and frequent contact with patients infected with the virus was declared by 20.4% and 14.7% of respondents, respectively.

In the depression subscale, the presence of moderate depressive symptoms was observed in 30.3% (M = 16.39 ± 2.48) of respondents, while severe and very severe depressive symptoms were reported in 16.2% (M = 23.44 ± 1.42) and 6.9% (M = 31.30 ± 3.49) of respondents, respectively. Moderate anxiety was found in 25.8% (M = 11.81 ± 1.58) of nurses, while high and very high levels of anxiety were reported in 16.2% (M = 17.3 ± 1.00) and 30.3% (M = 24.17 ± 4.63) of nurses, respectively. In the stress subscale, moderate and severe stress was reported by 35.4% (M = 21.02 ± 1.37) and 14.1% (M = 28.80 ± 2.59) of respondents, respectively (Table 1).

A significantly higher level of depressive symptoms (*p* = 0.045) and a higher level of anxiety (*p* = 0.009) were observed among women compared to men and among those living in urban agglomerations up to 50,000 inhabitants (depression *p* = 0.026; anxiety *p* = 0.009) compared to cities with > 50,000 population. Higher levels of depressive symptoms (*p* = 0.036) and anxiety (*p* = 0.041) were also found among those who graduated from medical secondary school/medical college compared to respondents with a bachelor’s or master’s degree in nursing. Significantly higher levels of anxiety, depression, and stress (*p* < 0.001) were shown by respondents with work experience between 21–30 years, nurses working in infectious hospital wards or those transformed into infectious units, and nurses employed in assisted living facilities. Also, significantly higher levels of symptoms of depression, anxiety, and stress (*p* < 0.001) were found among nursing staff who had very frequent or frequent contact with patients with suspected SARS-CoV-2 infection and COVID-19 patients. Only with regard to age, no statistically significant differences were found. The respondents were characterised by similar levels of symptoms of depression, anxiety, and stress throughout age subgroups (Table 2).

We performed a comparative analysis of the level of symptoms of depression, anxiety, and stress among nurses who had contact with COVID-19 patients and nurses who had no such contact. It was shown that those who had contact with COVID-19 scored significantly higher in the anxiety (*p* = 0.306) and stress (*p* = 0.001) subscales compared to those who had no contact with COVID-19 disease (Table 3).

The obtained results indicate that depression and anxiety are significantly positively correlated with the level of stress. Also, there is a strong positive correlation between the results in the depression and anxiety subscale (Table 4).

Three multiple regression analyses were performed to verify which of the assessed variables was a predictor of depressive symptoms, anxiety, and stress. Among the variables included in the analysis, contact with patients with suspected COVID-19 was a significant predictor of depressive symptoms. Significant predictors of anxiety included four variables: contact with COVID-19 patients, contact with patients with suspected COVID-19, workplace, and gender. Significant predictors of stress included two variables: contact with COVID-19 patients and contact with patients with suspected COVID-19 (Table 5, Table 6 and Table 7).

## 4. Discussion

The ongoing pandemic and the growing number of COVID-19 patients have given rise to increasing challenges for healthcare professionals. Unsafe working conditions can generate mental health problems such as stress, anxiety, and depression [18]. Although this problem affects all healthcare workers, nurses seem to be most affected. This is due to the fact that they are the largest group of healthcare professionals [10] and remain in the closest contact with patients who require constant care [26]. In this study, we assessed the prevalence of depressive symptoms, anxiety, and stress among nurses working in healthcare facilities during the COVID-19 pandemic. Our findings have raised concerns by suggesting that almost half of the surveyed nurses are affected by moderate (30.3%), severe (16.2%), and very severe (6.9%) symptoms of depression and stress (moderate—35.4%, severe—14.1%), and almost ¾ of respondents show moderate (25.8%), severe (16.2%) and very severe (30.3%) anxiety.

In most reports on mental health, even if all medical personnel were included in the study, nurses still accounted for the largest proportion of respondents [1]. Many authors observed the highest severity of anxiety and depression symptoms mainly in the most numerous professional group of nurses [27,28,29,30]. According to Lai et al., nurses (60.8% of the study group) were more likely to experience the symptoms of depression and anxiety compared to doctors. Severe and moderate symptoms of depression (according to scale PHQ-9 and GAD-7) were observed in 7.1% and 8.4% of nurses, respectively, and severe and moderate symptoms of anxiety were reported in 5.6% and 7.1% of nurses, respectively [28]. In their study among 221 nurses, conducted with the same tool we used in our study, Du et al. found moderate and severe symptoms of depression in 17.2%, anxiety in 31.2%, and stress in 10.9% of nurses [31]. The results we obtained in our study are higher than those presented above. However, the cited studies were conducted in China, mostly using different research tools, with different cut-off points, which may make accurate comparisons difficult. Furthermore, the above-mentioned studies were conducted in 2020, whereas our research took place a year later; therefore, it can be argued that the symptoms of depression, anxiety, and stress among nurses increase with the duration of the pandemic.

Assuming that such high scores obtained in this study may depend on the region in which the research was conducted, we decided to compare our findings with those obtained in different countries. In a previous study conducted in Poland (2020) (in Hospital Anxiety and Depression Scale HADS-A; HADS-D), which included nurses working in primary care (section of dermatology and nephrology), there were more nurses reaching the cut-offs for both anxiety (35.5%) and depression (17.7%) than other healthcare workers [32]. Comparing the research utilising the same tool we used in our study, we noticed high values for the symptoms of depression (39.5%), anxiety (50%), and stress (25.7%) in the group of nurses in the Nepal region, although still lower than in our study [33]. Also, researchers from England, France, Indonesia, Egypt, and Saudi Arabia point in their reports to mental health problems among nursing staff [34,35,36,37]. However, our study showed that the levels of depression, anxiety, and stress symptoms tend to be higher than in other countries. This may be explained by the fact that the pandemic is a new challenge for Polish nurses. As already mentioned, most studies come from Asian countries, where the previous SARS (2002–2003) and MERS (2012) pandemics may serve as a source of knowledge and experience, facilitating the implementation of appropriate preventive strategies [38]. Furthermore, it was the fourth wave of the ongoing pandemic in Poland, where infectious hospitals were again filled with COVID-19 patients, and the daily number of hospitalized patients ranged from about 12.000 up to about 17.000 people [39], while scheduled admissions in non-infectious hospitals were suspended and rooms or entire wards dedicated to emergency patients [40]. The deficit of nurses, although common all over the world, became particularly pronounced in Poland during the pandemic, becoming an additional problem [41]. For example, there are 5.1 nurses per 1000 inhabitants in Poland [42], with a mean age of a nurse of 52.6 years. [41]. Higher rates are observed in other countries, with the highest in Norway—18.1, followed by Switzerland—18.0, Iceland—15.7, and Germany—13.9 [42].

As for the potential factors affecting mental health, women, who account for the majority of nursing personnel [10], were most affected by the mental health crisis both before the pandemic [43] and after its outbreak [29,31,32,37,44,45]. Our study mostly included women (only eight men participated the study), and it was women (97.6% study group) who scored the highest for the symptoms of depression, anxiety, and stress, which should be interpreted with caution due to the small number of men. Lai et al. also demonstrated the highest levels of anxiety and depression among women (who accounted for 90.8% of the surveyed nurses) compared to men. Only the level of moderate anxiety was higher in the group of men [28].

In this study, frequent contact with patients suspected of being infected with SARS-CoV-2 was the predictor of depressive symptoms, while caring for COVID-19 patients was also a predictor of severe anxiety. Stress was associated with the two factors mentioned above. Work on the frontline, i.e., in an infectious hospital ward or a ward transformed into an infectious unit, and greater exposure to COVID-19 patients and/or patients suspected of being infected with the virus significantly escalated the symptoms of depression, anxiety and stress compared to nurses who had no direct contact or had limited contact with infected patients. These data are supported by Guo et al. and Lai et al., who found higher values for depression and anxiety symptoms among frontline vs. second-line medical staff. Also, compared with those working in tertiary hospitals, participants working in secondary hospitals were more likely to report moderate and severe symptoms of depression and anxiety [27,28]. Also in line with the observation of Liu et al., medical personnel directly involved in the treatment of COVID-19 patients in dedicated wards showed higher levels of depression and anxiety [30]. According to Cai et al., all frontline medical personnel experienced emotional stress during the pandemic, but it was the nursing personnel who felt more nervous and anxious compared to other groups [6]. These reports are supported by other sources [45,46]. In Germany, 55% of nurses in the COVID-19 ward, compared to just 12.5% in the regular ward, complained about stress at work [45]. Contrary to the cited studies, some authors found no correlation between the mental health of employees directly involved in the treatment of COVID-19 patients compared to medical personnel in other departments [36,47] and those who have direct contact with COVID-19 patients [31]. However, according to Buselli et al., working on the frontline has proven to be a potential risk factor for anxiety rather than depression [48].

As shown in our study, the levels of depression, anxiety, and stress symptoms were significantly higher in nurses with secondary education and longer work experience. Similar conclusions were drawn by Silwal et al. [33]. However, Choudhury et al. and Arafa et al. found no differences between these parameters [34,37]. In our study, age was not a factor significantly associated with the presence of symptoms of depression, anxiety, and stress, which corresponds with the results obtained by Du et al., who reported no correlation between either of the analysed parameters (symptoms of depression, anxiety, and stress) and age [31]. Some researchers have reported different values. Liang et al. found a correlation between age and mental health, with younger medical employees (≤30 years) showing higher scores for depressive symptoms than those >30 years of age [47]. Guo et al. and Arafa et al. also reported higher symptoms of depression and anxiety in younger personnel compared to older medical staff [27,37].

The deteriorating mental health of nurses during the pandemic may have serious consequences due to the negative impact on the quality of work [49], and thus on the quality of patient care. In addition, high levels of stress, excessive workload, and the resulting fatigue may be the cause of burnout among medical workers [50]. As emphasised by Vagni, resilience and hardiness are not highly effective protective factors [51]. This justifies the need to introduce psychological interventions, which, in the face of the global shortage of nurses, should be a priority of national health policies [52]. Unfortunately, some authors point to the lack of organisational support in terms of psychiatric help for nurses [53].

## 5. Conclusions

During the fourth wave of the pandemic, Polish nurses obtained high scores for depressive symptoms, anxiety, and stress levels. Nurses were found to be at immediate risk of developing mental health problems.

This study provides valuable data and indicates the need to pay attention to the mental health of nurses, especially those working on the frontline, in direct contact with patients with suspected SARS-CoV-2 infection, patients with COVID-19, and nurses with longer work service.

In our opinion, such high scores should be a valuable source of information for the management of medical facilities and contribute to the development of psychological support strategies to prevent further deterioration of mental health in nurses and to mitigate the impact of the pandemic on their psychological well-being.

Future research should focus on the long-term effects of the pandemic and develop targeted measures to prevent the deterioration of mental health of nurses.

Support is needed to reduce mental harm caused by the pandemic. Various interventions in the form of counseling, psychotherapy, and stress management programmes can be implemented. The workload can be reduced by recruiting more workers and providing opportunities to rest in the working environment, as well as by ensuring access to an appropriate amount of equipment and protective measures [54]. According to Greinacher, secondary traumatisation was associated with a lower level of social support [55].

### Study Limitations

This study has several limitations. These include a small sample size, which does not allow for data generalization, data collection limited to only one professional group of nurses, and using an online questionnaire, which could have influenced the number of responses sent. Comparison of study results by gender should not be interpreted unequivocally due to the small percentage of male participants in the study. Furthermore, the study had a cross-sectional design, which eliminated the possibility to assess the emotional state of respondents over time. Furthermore, differences between our findings and those presented by other authors may result from the use of different tools, which reduces the comparability of results. There is little research utilising the same tool in this area. Additionally, most of the studies with which we compared our findings were conducted at the onset of the pandemic, while our study was conducted during the fourth pandemic wave, at which point the pandemic-related psychological exhaustion may be greater. However, the survey may be helpful in developing a support strategy for nurses.

## Figures and Tables

**Table 1:** Depression, anxiety and stress in DASS-21—descriptive statistics.

	%	Mean	SD	MIN.	MAX.	Median
Depression subscale						
Normal (0–9)	27.9	5.24	2.70	0.00	8.00	6.00
Mild (10–13)	18.6	11.00	1.00	10.00	12.00	11.00
Moderate (14–20)	30.3	16.39	2.48	14.00	20.00	16.00
Severe (21–27)	16.2	23.44	1.42	22.00	26.00	24.00
Extremely Severe (28+)	6.9	31.30	3.49	28.00	36.00	30.00
Anxiety subscale						
Normal (0–7)	18.6	3.35	2.22	0.00	6.00	4.00
Mild (8–9)	9.0	8.00	0.00	8.00	8.00	8.00
Moderate (10–14)	25.8	11.81	1.58	10.00	14.00	12.00
Severe (15–19)	16.2	17.3	1.00	16.00	18.00	18.00
Extremely severe (20+)	30.3	24.17	4.63	20.00	36.00	24.00
Stress subscale						
Normal (0–14)	33.0	10.52	3.43	2.00	14.00	12.00
Mild (15–18)	17.4	17.27	0.97	16.00	18.00	18.00
Moderate (19–25)	35.4	21.02	1.37	20.00	24.00	20.00
Severe (26–33)	14.1	28.80	2.59	26.00	32.00	28.00
Extremely severe (34+)	0.0	-	-	-	-	-

**Table 2:** Sociodemographic variables and DASS-21 scores.

Parameter	Depression			Anxiety			Stress		
Sex									
	**M**	**SD**	**Z/F/*p***	**M**	**SD**	**Z/F/*p***	**M**	**SD**	**Z/F/*p***
Female	18.90	6.66	Z = 765.000 *p* = 0.045	14.68	8.06	Z = 597.000 *p* = 0.009	14.55	8.05	Z = 817.000 *p* = 0.072
Male	14.00	3.70		6.74	8.94		10.25	7.88	
Age									
20–30 years	14.83	8.32	F = 1.065 *p* = 0.374	14.16	7.95	F = 0.373 *p* = 0.828	17.52	6.81	F = 1.992 *p* = 0.095
31–40 years	13.05	7.25		13.75	7.74		17.72	7.54	
41–50 years	13.58	10.80		14.65	10.94		17.70	8.24	
51–60 years	15.40	5.42		15.31	5.30		18.47	3.71	
>61 years	16.00	5.16		14.80	11.08		23.40	2.31	
Place of residence									
Rural	11.86	5.39	F = 3.127 *p* = 0.026	10.55	6.21	F = 3.930 *p* = 0.009	18.13	6.32	F = 0.350 *p* = 0.789
Urban up to 50,000	16.53	5.69		16.68	6.93		18.62	4.80	
Urban between 51,000 and 100,000	15.75	11.64		14.65	12.62		17.30	9.87	
Urban > 100,000	13.90	8.04		14.33	7.44		17.92	6.41	
Education									
Medical high school/medical college	17.71	6.11	F = 3.362 *p* = 0.036	17.60	8.78	F = 3.235 *p* = 0.041	20.17	5.51	F = 2.694 *p* = 0.069
Bachelor of Nursing	13.93	7.94		14.35	7.31		17.50	6.45	
Master of Nursing	14.45	8.97		13.43	9.87		18.46	7.45	
Length of service in the profession									
<5 years	14.78	8.38	F = 8.316 *p* < 0.001	14.39	7.91	F = 6.119 *p* < 0.001	17.64	6.77	F = 7.945 *p* < 0.001
5–10 years	12.88	6.09		12.74	7.52		16.74	6.45	
11–20 years	9.47	8.17		10.81	7.94		14.61	8.17	
21–30 years	17.07	7.91		17.37	8.19		20.65	4.59	
>31 years	15.54	5.94		14.80	7.66		18.74	5.88	
Workplace									
Hospital, non-infectious ward	13.39	6.78	F = 5.832 *p* < 0.001	12.30	7.02	F = 17.811 *p* < 0.001	17.24	5.72	F = 5.832 *p* < 0.001
Hospital, ward transformed into infectious unit	16.45	8.04		18.14	6.95		20.13	5.86	
Infectious disease hospital	20.50	6.93		26.00	10.95		21.66	11.01	
Clinic	12.29	8.36		10.87	7.40		14.73	8.30	
Assisted living facility	21.14	3.02		24.00	0.00		21.71	0.75	
Other	13.52	6.06		13.88	9.41		18.23	7.27	
Do you have any contact with patients suspected of being infected with SARS-CoV-2?									
Yes, very frequently	18.78	9.07	F = 13.438 *p* < 0.001	20.87	8.11	F = 35.161 *p* < 0.001	21.70	6.87	F = 19.737 *p* < 0.001
Yes, frequently	14.40	6.77		14.74	6.52		18.86	4.67	
Yes, occasionally	12.25	7.84		10.67	7.24		15.25	6.91	
No, never	11.50	3.50		11.83	5.03		16.25	4.01	
Do you have any contact with COVID-19 patients?									
Yes, very frequently	18.97	9.53	F = 15.737 *p* < 0.001	21.94	7.96	F = 45.895 *p* < 0.001	22.41	6.82	F = 18.597 *p* < 0.001
Yes, frequently	17.02	6.57		17.87	5.39		19.51	4.69	
Yes, occasionally	11.90	6.67		10.92	6.17		16.54	6.17	
No, never	13.57	7.88		12.34	7.99		15.68	6.33	

Z—Mann-Whitney U; F—Anova; *p*—statistical significance.

**Table 3:** Differences in the level of depression, anxiety and stress depending on exposure to COVID-19 disease.

Variable	No Contact with COVID-19		Contact with COVID-19		t(df)	*p*
	M	SD	M	SD		
Depression	13.57	7.88	14.68	8.11	−1.025 (331)	0.306
Anxiety	12.34	7.99	15.06	8.12	−2.499 (331)	0.013
Stress	15.68	6.33	18.61	6.58	−3.334 (3310	0.001

M—arithmetic mean, SD—standard deviation, t—t-Student value, df—degrees of freedom, *p*—level of significance.

**Table 4:** Correlation analysis for stress, depression, and anxiety.

Variable	Stress		Depression		Anxiety	
	r	*p*	r	*p*	r	*p*
Stress	-	-	-	-	-	-
Depression	0.778	<0.001	-	-	-	-
Anxiety	0.725	<0.001	0.791	<0.001	-	-

r—Pearson’s r correlation coefficient.

**Table 5:** Multiple regression analysis for the variable depression predicting the likelihood of depression.

Variable	Depression Adjusted R^2^ = 0.101; F(7, 325) = 6.34; *p* < 0.001				
	*b*	Error	*β*	*t*	*p*
Age	−1.97	1.11	−0.31	−1.778	0.076
Place of residence	−0.44	0.41	−0.06	−1.068	0.286
Education	−0.56	0.79	−0.04	−0.705	0.481
Years of service	1.47	0.99	0.26	1.488	0.138
Place of residence	−0.46	0.39	−0.07	−1.176	0.241
Contact with a patient suspected of being infected with SARS-CoV-2	1.97	0.73	0.22	2.684	0.008
Contact with COVID patient	0.62	0.66	0.08	0.927	0.355

*R^2^*—coefficient of determination, *F*—Fisher’s test value, *b*—non-standardised regression coefficient, *Error*—standard error, *β*—standardised regression coefficient, *t*—Student’s t-test value, *p*—significance level.

**Table 6:** Multiple regression analysis for the anxiety variable predicting the likelihood of anxiety.

Variable	Anxiety Adjusted R^2^ = 0.249; F(7, 325) = 16.71; *p* < 0.001				
	*b*	*Błąd*	*β*	*t*	*p*
Age	−0.52	1.03	−0.08	−0.508	0.612
Place of residence	−0.05	0.38	−0.01	−0.118	0.906
Education	−0.98	0.73	−0.07	−1.354	0.177
Years of service	0.03	0.91	0.01	0.033	0.973
Place of residence	−0.85	0.36	−0.12	−2.361	0.019
Contact with a patient suspected of being infected with SARS-CoV-2	1.86	0.68	0.21	2.738	0.007
Contact with COVID patient	2.20	0.61	0.28	3.583	<0.001

*R^2^*—coefficient of determination, *F*—Fisher’s test value, *b*—non-standardised regression coefficient, *Error*—standard error, *β*—standardised regression coefficient, *t*—Student’s t-test value, *p*—significance level.

**Table 7:** Multiple regression analysis for the stress variable predicting the likelihood of stress.

Variable	Stress Adjusted R^2^ = 0.141; F(7, 325) = 8.78; *p* < 0.001				
	*b*	*Błąd*	*β*	*t*	*p*
Age	−0.35	0.89	−0.07	−0.396	0.692
Place of residence	−0.30	0.33	−0.05	−0.903	0.367
Education	0.38	0.63	0.03	0.606	0.545
Years of service	0.38	0.79	0.08	0.472	0.637
Place of residence	−0.36	0.31	−0.06	−1.143	0.254
Contact with a patient suspected of being infected with SARS-CoV-2	1.39	0.59	0.19	2.361	0.019
Contact with COVID patient	1.26	0.53	0.20	2.365	0.019

*R^2^*—coefficient of determination, *F*—Fisher’s test value, *b*—non-standardised regression coefficient, *Error*—standard error, *β*—standardised regression coefficient, *t*—Student’s t-test value, *p*—significance level.

## Data Availability

The datasets generated and/or analyzed during the current study are not publicly available due to confidentiality, but data is accessible from the corresponding author on reasonable request.
